# An exploratory pilot study of integrated mini-CEX and DOPS assessments in general medicine residency: feasibility and longitudinal outcomes

**DOI:** 10.1016/j.clinsp.2026.101005

**Published:** 2026-07-20

**Authors:** Shanshan Pang, Xia Li, Sheng Cheng, Li Qin, Jiuyan Ding, Wenchun Song, Jing Zhou

**Affiliations:** a Department of General Medicine, Yancheng Third People's Hospital, the affiliated hospital of Jiangsu Medical College, Yancheng, China; b Department of General Medicine, Yancheng Third People's Hospital, Affiliated Hospital 6 of Nantong University, Yancheng Third People's Hospital, Yancheng, China; c Department of Pediatrics, Affiliated Hospital 6 of Nantong University, Yancheng Third People's Hospital, Yancheng 224000, China

**Keywords:** Clinical proficiency, Adaptive performance, Mini-CEX, DOPS, General medicine

## Abstract

**Objective:**

This exploratory pilot study aims to investigate the practices for enhancing clinical competence and exploring adaptive performance in General Medicine residents. It focuses on providing initial insights into how integrated assessment tools and structured feedback mechanisms are associated with the progression of clinical skills in a specific residency setting.

**Methods:**

A total of 30 General Medicine residents were selected for this single-arm longitudinal investigation. Subjects underwent standardized training at the General Medicine base of the Sixth Affiliated Hospital of Nantong University. Competency progression was assessed using a combination of Mini-CEX and DOPS. Each resident underwent three assessments at baseline (Month-1), mid-term (Month-4), and end (Month-7), with structured feedback provided by six calibrated senior attending physicians.

**Results:**

Significant improvements were observed across the three assessment stages in both Mini-CEX and DOPS scores (*p* < 0.0001). Repeated-measures ANOVA indicated a robust upward trajectory in clinical and procedural competencies over the seven-month training period.

**Conclusion:**

The integration of Mini-CEX and DOPS within a boundary-learning framework is associated with significant improvements in residents' clinical consultation and procedural proficiency. While these preliminary findings suggest that the structured feedback mechanism supports the development of core competencies, further research using validated psychometric scales is required to definitively measure the transition from routine proficiency to adaptive expertise.

## Highlights

• Integrating Mini-CEX and DOPS enhances clinical skills in General Medicine Residency.
• Boundary-learning framework bridges specialist and generalist expertise.
• Structured feedback supports the transition to adaptive clinical performance.
• A pilot study on feasible integrated assessments for medical education.

## Background

General Medicine education aims to cultivate physicians capable of navigating complex healthcare settings through a mastery of both medical knowledge and adaptive problem-solving skills.^1^^,^^2^ These physicians must possess high-level clinical skills as well as strong innovative thinking and problem-solving abilities.^3^ However, traditional medical assessment methods often focus on evaluating knowledge and skills, which typically fail to fully reflect the residents' actual performance and innovative capabilities in clinical environments.^4^ General Medicine physicians must demonstrate clinical skills, communication, teamwork, and critical thinking, which are hard to assess through traditional exams. Assessing innovation and problem-solving abilities is a major challenge for educators.^5^ Boundary-learning as an innovative educational framework goes beyond traditional knowledge transmission. It is defined as a dynamic process of interaction, reflection, and adjustment that occurs when learners cross professional, disciplinary, or practical boundaries. Unlike Situated or Interprofessional Learning, Boundary-learning focuses on the cognitive growth triggered by “boundary crossing”, such as transitioning between generalist and specialist perspectives. In General Medicine, where physicians must navigate complex healthcare settings and solve multifaceted problems, this framework explains how interaction between boundaries stimulates innovative thinking and adaptive problem-solving.^6^^,^^7^ In recent years, boundary-learning has gained attention as an innovative learning model in medical education. The boundary-learning framework emphasizes the dynamic, interactive, and reflective nature of learning. It asserts that education is not merely the transmission of knowledge but an ongoing process of interaction, reflection, and adjustment.^8^ By emphasizing the interaction between residents and instructors, as well as between residents and clinical practice, boundary-learning fosters self-awareness and practical skills in learners. This perspective offers a new approach to medical education, particularly in the training of innovative General Medicine talent. Boundary-learning helps residents adapt to changing clinical environments and enhances problem-solving through continuous feedback and adaptable teaching strategies.^9^ Unlike traditional reflective practice, which typically focuses on the individual's reflection on their own practice, aiming to enhance learning outcomes through increased self-awareness and analysis of experiences, experiential learning places more emphasis on gaining experience through direct participation in practical activities, and learning through those experiences, highlighting perception and experience in practice. In contrast to these theories, “boundary-learning” focuses on the dynamic interaction across domains. It views learning not only as a process of knowledge transfer but also as a continuous process of reflection, adjustment, and interaction. In this framework, the interaction between the learner, the mentor, and clinical practice becomes the core of education. Through continuous feedback and flexible adjustments in teaching strategies, students are better able to adapt to the complex and ever-changing clinical environment, thereby fostering the development of innovative thinking. Therefore, “boundary-learning” not only emphasizes individual reflection or the accumulation of experience but also focuses on how the interaction between boundaries across different domains can stimulate learners' innovative potential and problem-solving abilities.

To address the challenges in medical education assessment, Mini-CEX (Mini Clinical Evaluation Exercise) and DOPS (Direct Observation of Procedural Skills) have emerged as new assessment tools and are widely applied in medical education.^10^ Mini-CEX is a comprehensive assessment tool for clinical skills, capable of evaluating residents' clinical abilities in multiple areas such as history taking, clinical judgment, physical examination, and communication skills.^11^ It uses a brief evaluation method, observing and assessing residents' clinical performance to help them understand their strengths and weaknesses in a timely manner, provide personalized feedback, and guide their further development.^12^ DOPS, on the other hand, is more focused on assessing procedural skills. It directly observes residents' performance in hands-on procedures, evaluating their professional skills in techniques such as surgery, injections, catheter insertion, and other medical procedures.^13^ The DOPS tool emphasizes direct observation on-site, allowing mentors to gain a clearer understanding of the details and standardization of the residents' technical performance, and subsequently offer improvement suggestions.^14^ Combining Mini-CEX and DOPS creates a comprehensive evaluation system that assesses both theoretical knowledge and practical clinical skills. These tools not only assess residents' clinical skills but also enable instructors to identify residents' strengths and weaknesses in clinical practice through multiple direct observations and feedback.^15^ This assessment method is highly flexible, allowing for real-time adjustments to teaching strategies based on residents' specific performance, ensuring that they continue to improve their clinical abilities, especially in complex clinical judgment and procedural skills.^16^

This study explores a novel educational approach: integrating Mini-CEX and DOPS within a boundary-learning framework to foster clinical competence and adaptive expertise in General Medicine residents. In this context, “boundary-learning” represents the cognitive and professional growth occurring at the intersection of holistic generalist reasoning and high-precision specialized procedures. By operationalizing Mini-CEX to assess the consultative boundary and DOPS for the procedural boundary, these tools transcend mere evaluation; they act as catalysts for real-time feedback and reflection between residents, instructors, and clinical practice. This interactive process enables residents to identify skill gaps and solve complex clinical problems, while allowing instructors to adapt teaching strategies dynamically based on performance. As an exploratory, prospective educational study, the authors aim to demonstrate how this unified framework can stimulate innovative potential and prepare residents for future medical challenges. To ensure methodological transparency, the study adheres to the STROBE Statement for observational reporting and the SQUIRE-EDU guidelines for quality improvement in medical education.

## Materials and methods

### Definitions of key terminology

In this study, the term “highly skilled and innovative medical professionals” is operationally defined based on the integration of clinical proficiency and adaptive expertise within the General Medicine context:

Highly Skilled: Refers to residents who achieve a “Satisfactory” level or higher (score ≥6 on the 9-point Likert scale) across all seven dimensions of the Mini-CEX and all six dimensions of the DOPS, demonstrating independent procedural and diagnostic competence.

Innovative (Adaptive Performance): In the context of this residency training, “innovation” is operationally defined as adaptive clinical performance ‒ the ability to synthesize interdisciplinary knowledge to manage complex clinical “boundaries”. The authors acknowledge that high scores (≥7) in complex domains (such as “Clinical Judgment” and “Ability to deal with complications”) serve as suggestive proxy indicators of adaptive expertise, reflecting a high level of clinical maturity rather than the invention of novel medical protocols.

### Study design and participants

The study employed a prospective, single-group longitudinal design involving 30 General Medicine residents. To monitor the progression of clinical competencies, assessments were categorized into three sequential phases based on training duration: Group A (Baseline) at Month-1, Group B (Mid-term) at Month-4, and Group C (Final) at Month-7. This design allowed each participant to serve as their own historical control, facilitating the tracking of individualized skill acquisition over time. While assessments were conducted by trained mentors, the study primarily focused on the longitudinal progression of residents' skills under this comprehensive feedback framework. To ensure the authenticity of clinical performance, residents were evaluated during routine clinical encounters. While residents were aware of being assessed for feedback purposes, the specific scoring focus of each session was not disclosed beforehand to minimize performance bias. However, the authors acknowledge that a more rigorous study design would include a comparison group (e.g., standard assessment vs. combined tools) to more accurately attribute observed outcomes to the intervention and avoid potential confounding factors, such as the natural progression of clinical skills over time. While the absence of a control group limits the ability to directly attribute the improvements solely to the intervention, the authors believe the results provide valuable preliminary insights into the effectiveness of this combined approach. Future studies should consider incorporating a comparison group to strengthen the validity of the findings. With the aim of exploring how the Mini-CEX and DOPS assessment tools can enhance teaching quality during the early development of highly skilled and innovative medical professionals in General Medicine. The implementation of Mini-CEX and DOPS was explicitly guided by the boundary-learning framework. Mini-CEX served as a tool to evaluate “interactional competence” across disciplinary boundaries, while DOPS assessed the “procedural adaptation” of residents in multidisciplinary clinical scenarios. These assessments acted as “boundary objects” ‒ facilitating a shared reflective space between the mentor and the resident to bridge the gap between theoretical knowledge and complex clinical practice. The integration of Mini-CEX and DOPS was designed to map the residents' progression across the learning boundary. While Mini-CEX evaluates the consultative boundary (e.g., patient-centered communication and holistic reasoning), DOPS captures the technical boundary (e.g., procedural mastery). This dual-assessment approach ensures that residents are not merely siloed into technical tasks but are learning to synthesize specialist skills within the generalist identity. Furthermore, this method aims to promote the reform and innovation of General Medicine education. The study participants consisted of 30 General Medicine residents, all selected from the General Medicine Department of the Sixth Affiliated Hospital of Nantong University. These residents varied in age and clinical experience and represented different year levels to ensure that the assessment method would be widely applicable across a diverse group of participants. These residents will complete a 7-month rotation in the General Medicine Department, during which they will undergo routine clinical training and participate in various clinical procedures and medical record management tasks. To simulate a realistic clinical environment as closely as possible, all residents will undergo comprehensive clinical internships under the guidance of their mentors, covering multiple aspects such as disease diagnosis, treatment, clinical skills, and doctor-patient communication. Prior to the study commencement, all 30 General Medicine residents were fully informed of the study's objectives, methods, and the voluntary nature of their participation. Written informed consent was obtained from all participating residents. Participation was voluntary, and it was assured that their decision to participate or withdraw would not affect their standardized training progress or evaluation results. Furthermore, all collected data, including assessment scores and feedback responses, were anonymized and maintained with strict confidentiality to protect the privacy of the participants, in compliance with ethical guidelines for human subjects in education research.

### Research methods

During the rotation, each resident will engage in clinical work under the supervision of a mentor, who is responsible for patient management and providing professional guidance. To enhance the clinical skills of the residents, weekly small lectures and case discussions will be arranged, along with regular training in physical examination techniques, interpretation of auxiliary examinations, and basic procedural skills. The Mini-CEX and DOPS assessments will be conducted at the beginning, mid-term, and end of the training. The assessment content includes medical interviews, physical examination, clinical judgment, technical skills, communication abilities, and humanistic care. The evaluations will use a 9-point scale: 1‒3 points indicating unsatisfactory performance, 4‒6 points indicating satisfactory performance, and 7‒9 points indicating excellent performance. After each assessment, instructors will provide detailed feedback to the residents, highlighting their strengths and areas for improvement, and offering one-on-one guidance to help them further develop their skills. Boundary learning emphasizes the integration of knowledge and skills across different disciplines or fields. Mini-CEX can assess students' clinical communication, decision-making, and multidisciplinary collaboration skills in cross-disciplinary contexts, helping students make connections across various disciplines. For example, during case discussions in different disciplinary contexts or collaboration in interdisciplinary teams, Mini-CEX can evaluate how students integrate knowledge from different fields, thereby enhancing their cross-disciplinary thinking and practical skills. The real-time feedback provided by Mini-CEX helps students identify their strengths and areas for improvement. In boundary learning, students often face complex and blurry disciplinary boundaries. Mini-CEX can provide feedback on cross-disciplinary interactions, helping students make more insightful judgments and decisions when dealing with unclear boundaries or multicultural settings. Boundary learning also emphasizes students' ability to adapt to different cultural, linguistic, and disciplinary contexts. Through Mini-CEX, teachers can assess how students cope with diverse learning environments, gaining insight into their adaptability and their interactions with multidisciplinary teams. In boundary learning, students need to perform specific tasks or procedures within multidisciplinary collaborations. DOPS can be used to assess students' procedural skills in different disciplinary environments. For example, during joint surgeries or diagnostic processes in interdisciplinary teams, DOPS can observe how students collaborate with experts from other fields, assessing their ability to cooperate and perform in multidisciplinary environments. Boundary learning emphasizes students' adaptability in different cultural or disciplinary settings. DOPS helps teachers understand whether students can flexibly adjust their approach to tasks in various disciplinary backgrounds and team environments by observing their performance in specific situations. DOPS also provides real-time feedback on the procedural process, helping students recognize the challenges and opportunities of performing specific tasks in different disciplinary contexts. In the context of boundary learning, this feedback extends beyond technical skills to include communication and collaboration with experts from other fields. By combining Mini-CEX and DOPS, teachers can comprehensively assess students' overall abilities in boundary learning environments. For example, when students perform complex procedures within a multidisciplinary team, they need not only to demonstrate procedural skills (assessed by DOPS) but also to communicate, coordinate, and make decisions effectively with team members (assessed by Mini-CEX). This combined assessment approach provides a more comprehensive reflection of students' core abilities in multidisciplinary collaboration and boundary learning. Furthermore, through this new combination, teachers can better evaluate how students creatively solve problems in cross-disciplinary contexts. Students' procedural skills, communication abilities, and integration of cross-disciplinary knowledge are all fully captured in this comprehensive assessment.

**Fig. 1:**
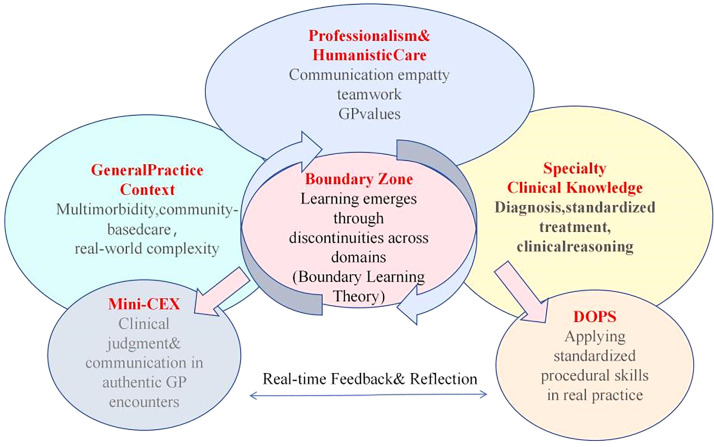
Conceptual framework of boundary-learning in the early development of innovative General Medicine professionals. (The “Boundary Zone” acts as the nexus where General Practice context, Specialty knowledge, and Professionalism intersect. Learning emerges through the discontinuities in these areas, with Mini-CEX and DOPS serving as evaluative mechanisms that facilitate real-time feedback and reflection, ultimately fostering innovative clinical competence).

Operationalization of Boundary-Learning Tools: To operationalize the boundary-learning framework, Mini-CEX and DOPS were employed not merely as scoring sheets but as “boundary objects” that facilitate cognitive crossing between generalist and specialist domains. Mini-CEX as a Consultative Boundary Object: It was used to bridge the gap between abstract generalist reasoning (holistic care) and concrete specialist knowledge (disease-specific diagnosis) during patient encounters. By focusing on “Clinical Judgment” and “Communication”, the tool forced a synthesis of these two often-siloed perspectives. DOPS as a Procedural Boundary Object: It acted as a nexus between standardized technical protocols and the unpredictable real-world General Medicine setting. Evaluators used DOPS to observe how residents adapted rigid specialist procedures to meet the specific needs of multi-morbid patients in a generalist ward. Structured Reflective Space: During the 15‒20 min feedback sessions, the physical assessment forms served as a shared reflective platform. The “feedback sandwich” was specifically structured to address the “discontinuities” identified at these boundaries, encouraging residents to transition from routine protocol-following to adaptive problem-solving (Fig. 1). To operationalize the boundary-learning framework, Mini-CEX and DOPS were utilized as “boundary objects” to bridge the gap between fragmented specialist knowledge and holistic generalist practice. For instance, during a Mini-CEX assessment involving a patient with Chronic Obstructive Pulmonary Disease (COPD) complicated by heart failure, the mentor did not merely score the diagnostic accuracy. Instead, the feedback focused on the “boundary crossing” between respiratory and cardiac guidelines, forcing the resident to synthesize a unified management plan. Similarly, in DOPS sessions for thoracentesis, the evaluation transcended sterile technique to observe how residents adapted standardized protocols for frail, multi-morbid patients. This process transformed the assessment from a static checklist into a dynamic dialogue where the physical form served as a shared platform for identifying knowledge “discontinuities”.

### Evaluation tools

The Mini-CEX (Mini Clinical Evaluation Exercise) and DOPS (Direct Observation of Procedural Skills) forms were used to assess the clinical and procedural skills of the residents. These forms are structured to capture various aspects of performance, including history taking, clinical judgment, physical examination, communication skills, and technical procedure proficiency.

Mini-CEX Evaluation Form (Table 1): This form assesses a resident's clinical skills in real-time, focusing on their interaction with patients, communication, and clinical reasoning. DOPS Evaluation Form (Table 2): This form is specifically used for evaluating procedural skills, where assessors directly observe residents performing medical procedures such as injections, catheter insertion, and basic surgical techniques. Similar to the Mini-CEX, the DOPS form uses a rating scale to assess proficiency and technique. The 9-point Likert scale was defined with specific clinical performance anchors: Scores 1–3 (Unsatisfactory): The resident demonstrated significant gaps in core competencies, requiring constant intervention or failing to ensure patient safety. Scores 4–6 (Satisfactory/Competent): The resident performed the task independently and safely, meeting the expected standard for their level of training. Scores 7–9 (Excellent/Superior): The resident demonstrated highly nuanced clinical judgment or technical mastery exceeding expectations for their current level. Participants were categorized into three groups based on the assessment timeline to track longitudinal progression: Group A represents the baseline assessment at the beginning of the rotation (Month-1), Group B represents the mid-term assessment (Month-4), and Group C represents the final assessment at the end of the 7-month training period (Month-7). A total of six senior attending physicians or higher from the General Medicine Department served as evaluators. To ensure longitudinal consistency, the same pool of evaluators was used for all baseline (Group A), mid-term (Group B), and final (Group C) assessments. Each evaluator underwent a four-hour standardized calibration workshop, which included: 1) A comprehensive review of the Mini-CEX and DOPS scoring rubrics and anchor definitions; 2) Independent scoring of filmed resident-patient encounters; and 3) A group consensus session to resolve scoring discrepancies, particularly those exceeding 1-point. This rigorous process ensured high inter-rater reliability, as evidenced by the achieved ICC and Kappa values (Table 3).

**Table 1:** Mini-CEX evaluation.

Criterion	Score (1‒9)	Comments
Patient history taking		
Physical examination		
Clinical reasoning/judgment		
Communication skills		
Overall performance

**Table 2:** DOPS evaluation form.

Procedure	Score (1‒9)	Comments
Sterile technique		
Procedural skill proficiency		
Patient safety/comfort		
Overall performance

**Table 3:** Summary of evaluator characteristics and statistical metrics.

Characteristic / Domain	Detail / Value
Evaluator Profile	
Number of evaluators	6
Professional Title	Senior Attending Physician or higher
Mean years of clinical experience	12.5 years (Approximate)
Training Details	
Workshop Duration	4 h
Training Components	Rubric review, video-based scoring, consensus sessions
Inter-Rater Reliability (IRR)	
Mini-CEX Overall ICC	0.87 (95% CI 0.80‒0.91)
DOPS Overall ICC	0.83 (95% CI 0.75‒0.89)
Overall Cohen’s Kappa	0.80
Statistical Implementation	
Primary Inferential Test	Repeated-measures ANOVA
Post-hoc Analysis	Bonferroni correction

### Evaluator training and standardization

To minimize subjectivity and ensure the consistency of evaluation, all evaluators participated in a standardized calibration training prior to the implementation of Mini-CEX and DOPS. Specifically, the four-hour calibration workshop for mentors included a dedicated module on identifying “boundary discontinuities”. Mentors were trained not only to score clinical accuracy but to explicitly facilitate “boundary-crossing” conversations when a resident’s performance stagnated between generalist reasoning and specialist procedures. The training included detailed explanations of the scoring criteria, performance indicators, and feedback procedures. During the training, evaluators reviewed sample cases and discussed rating discrepancies to align their understanding of the assessment standards. Regular meetings were also conducted throughout the study to review evaluation outcomes and recalibrate scoring when necessary. This process aimed to enhance inter-rater reliability and ensure fairness and objectivity in the assessment results.

### Feedback process

The feedback process was structured and provided to each resident after every Mini-CEX and DOPS assessment. Feedback was delivered orally during one-on-one meetings between the evaluator and the resident, ensuring immediate clarification of strengths and areas for improvement. Additionally, written feedback was provided via a standardized feedback form, which included specific comments on performance and recommendations for further development. Each feedback session lasted approximately 15‒20 minutes, allowing sufficient time for both verbal discussion and for the resident to ask questions and clarify doubts. Feedback was delivered in a timely manner, immediately after each assessment, to maximize its effectiveness and to facilitate real-time improvement in clinical skills. Regular follow-up feedback sessions were also scheduled midway through the training to assess progress and adjust learning goals as needed. To ensure the quality and consistency of feedback, all evaluators followed the “feedback sandwich” technique (highlighting strengths, identifying areas for improvement, and providing an action plan). During the initial calibration workshop, evaluators practiced delivering constructive feedback based on sample video cases to align their communication styles. Furthermore, the lead investigator periodically reviewed the written comments on a random sample of assessment forms to ensure they were specific, actionable, and aligned with the boundary-learning framework. The boundary-learning framework actively shaped the structured feedback sessions in several ways that distinguish this intervention from standard assessment-feedback routines: Identification of Discontinuities: Mentors were instructed to use the Mini-CEX and DOPS forms as “boundary objects” to visually highlight the gaps (discontinuities) between a resident's specialized procedural knowledge and the complex, multi-morbid reality of general practice. Prompting Reflective Negotiation: Rather than just providing corrective guidance, mentors utilized “boundary-crossing prompts” (e.g., “How does this specialist protocol conflict with the patient's community-based care needs?”). This forced residents to engage in “reflective negotiation” to bridge the generalist-specialist divide. Collaborative Action Planning: The action plan (the final part of the “feedback sandwich”) was co-constructed to focus specifically on adaptive strategies for future boundary encounters, ensuring that feedback translated into measurable clinical adaptation.

### Data analysis

Data were analyzed using SPSS 26.0 software. Measurement data were expressed as mean ± standard deviation (x ± s). To account for the non-independent nature of repeated measures from the same subjects, competency progression across the three phases (baseline, mid-term, and final) was analyzed using repeated-measures ANOVA as the primary statistical test. Post-hoc comparisons were performed using the Bonferroni correction to identify specific differences between time points. The effect size (Cohen’s *d*) for the overall training impact was calculated to assess the practical significance of clinical competence gains beyond mere statistical significance (p-values). Data visualization was carried out using GraphPad 8.0 software. To ensure the consistency and reliability of the evaluations, inter-rater reliability was calculated using the Intraclass Correlation Coefficient (ICC) for continuous variables and Cohen’s Kappa for categorical assessments. These reliability metrics were confirmed through standardized evaluator calibration to ensure measurement stability.

Qualitative data, comprising 30 individual Self-Reflection Notes and written comments from evaluator forms, were analyzed using thematic analysis. The analytic process followed Braun and Clarke’s six-step framework: 1) Data familiarization; 2) Initial code generation; 3) Theme searching; 4) Theme review; 5) Defining and naming themes; and 6) Final reporting. To ensure rigor, two researchers independently performed inductive coding, with any disagreements resolved through iterative discussion and, when necessary, adjudication by a third senior researcher to reach a negotiated consensus on the final thematic framework. This process focused on reconciling different interpretive perspectives to enhance the trustworthiness and depth of the qualitative findings, rather than enforcing a singular view. To enhance trustworthiness, member checking was performed with a subset of participants (*n* = 5) to confirm that the derived themes accurately captured their learning experiences. Residents' perceptions were collected via a structured questionnaire employing a Likert scale. These responses were recorded as percentages to capture feedback on assessment effectiveness, feedback timeliness, and impact on the learning experience.

## Results

### Comparison of mini-CEX scores between groups A, B, and C

**Fig. 2:**
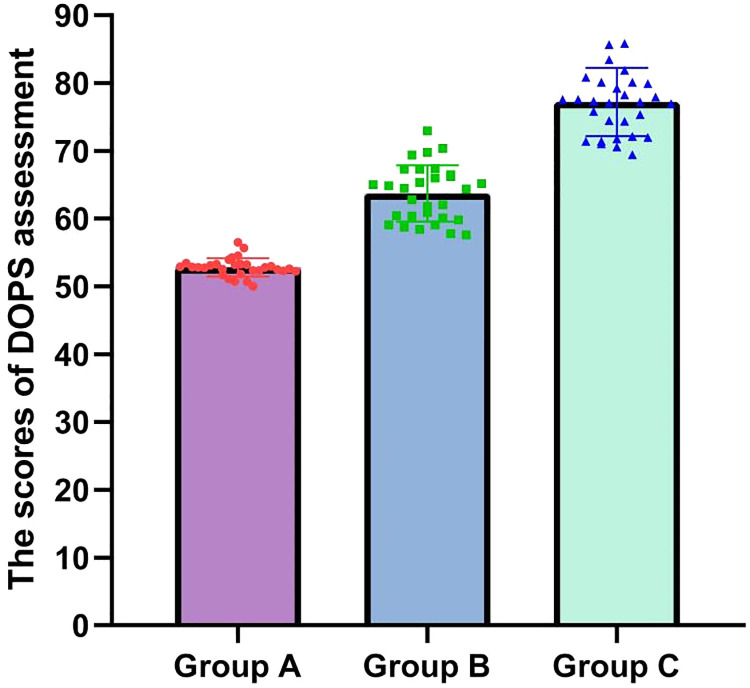
Comparison of Mini-CEX Scores Between Groups A, B, and C (Y-axis shown on a full scale of 0–60 to ensure visual transparency of the competency progression).

The Mini-CEX scores for Groups A, B, and C were (34.85 ± 1.34), (42.67 ± 2.77), and (52.29 ± 1.79), respectively. Significant differences were observed between the three groups (*p* < 0.001) (Fig. 2).

### Comparison of DOPS scores between groups A, B, and C

**Fig. 3:**
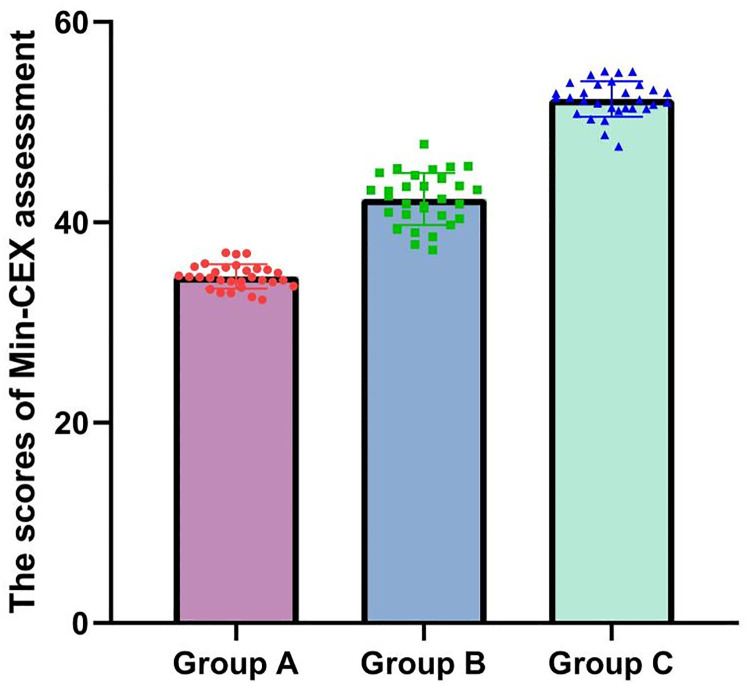
Comparison of DOPS Scores Between Groups A, B, and C (Y-axis shown on a full scale of 0–100 to accurately represent the magnitude of procedural skill acquisition).

The DOPS scores for Groups A, B, and C were (52.85 ± 1.49), (64.10 ± 4.05), and (75.74 ± 5.31), respectively. Repeated-measures ANOVA revealed significant improvements in procedural competency across the three assessment phases, with post-hoc Bonferroni tests confirming significant differences between each pair of time points (*p* < 0.01) (Fig. 3).

### Feedback from residents

The majority of residents held a positive view of the integrated assessment and feedback process. Quantitative survey results indicated that 94% of the residents believed the feedback was constructive and provided more learning opportunities. Furthermore, 80% of the residents considered Mini-CEX and DOPS to be effective teaching tools that enhanced their procedural skills and provided a platform to express their clinical views. Regarding the educational environment, 67% of the participants felt the feedback timing was sufficient, and 60% believed these assessment tools contributed to an improved teacher-student relationship. Additionally, 74% of the residents reported that being observed during clinical encounters did not negatively affect their performance.

Qualitative analysis of the feedback forms and the 30 individual Self-Reflection Notes revealed three recurring themes that illustrate the progression of adaptive expertise within the boundary-learning framework (detailed in Table 4):

**Table 4:** Qualitative themes extracted from evaluator feedback and resident reflections.

Core Theme	Description	Typical Evidence (Revised for Rigor)
Interdisciplinary Integration	Ability to apply and synthesize integrated specialist knowledge within a holistic generalist framework.	Residents demonstrated the ability to reconcile disparate specialist guidelines to create a unified management plan for multi-morbid patients.
Adaptive Behavioral Adjustment	Transition from rigid protocol adherence to active, real-time adjustments based on clinical feedback.	Residents showed flexible handling of complications or unexpected variations during procedures, moving beyond rote technical execution.
Communication Nuance	Strengthening of patient-centered awareness and professional humanistic care at complex boundaries.	Improved ability to adapt medical language and empathetic communication for elderly patients with multiple comorbidities and high care complexity.

Theme 1: Interdisciplinary Integration. Residents noted that the integrated feedback helped them “bridge the gap” and synthesize specialist technical knowledge within a holistic generalist management plan.

Theme 2: Real-time Self-Correction. The structured feedback served as a catalyst for immediate behavioral change, allowing residents to transition from rigid protocol adherence to active adjustments in sterile techniques and clinical judgment.

Theme 3: Communication Nuance. Mentors emphasized the use of patient-centered language at complex clinical boundaries, which residents perceived as essential for internalizing professional humanistic care.

To ensure the rigor and trustworthiness of these findings, the qualitative analytic process focused on reaching a negotiated consensus among the research team. Any differences in initial coding or theme interpretation were resolved through iterative discussion and, when necessary, adjudication by a third senior researcher. This process aimed to reconcile diverse interpretive perspectives and achieve an agreement on the final thematic framework that accurately reflected the residents' learning experiences. Following each oral feedback session, residents completed a brief Self-Reflection Note summarizing their key takeaways and outlining a specific learning plan for the subsequent training phase. These reflections provided a longitudinal record of the residents' evolving critical thinking and problem-solving abilities as they progressed through the seven-month rotation.

## Discussion

The development of clinical proficiency and adaptive expertise in General Medicine residents is associated with a synergistic approach encompassing clinical skills, adaptive thinking, and humanistic care. This study explored the integration of Mini-CEX and DOPS within a “boundary-learning” framework to address these complex educational needs.

### Assessment synergy: integrating mini-CEX and DOPS

Traditional assessment methods often focus on single-dimensional knowledge acquisition, which fails to capture the complexity of residency training. The present results demonstrate a significant longitudinal progression in clinical competencies, suggesting that the integration of Mini-CEX and DOPS creates a robust evaluation framework capable of capturing the multifaceted nature of General Medicine training. The significant improvement observed across the three stages (*p* < 0.001) reflects the effective capture of competencies through this dual-assessment approach.^11^ While Mini-CEX is effective for assessing “soft skills” like reasoning and communication, the addition of DOPS provides rigorous observation of procedural proficiency. This synergy allows mentors to identify weaknesses in both diagnostic logic and technical execution in real-time, providing a more holistic profile of the resident than either tool could offer in isolation.

### Feedback mechanisms and competence internalization

A key finding of this research is the substantial impact of the structured feedback mechanism. The significant score improvements observed in this study underscore the potency of real-time, structured feedback. Unlike traditional end-of-rotation evaluations, this framework facilitates immediate remediation through the “feedback sandwich” technique and standardized scoring anchors. The robust progression in assessment scores over the seven-month training period reflects an “internalization” process, where residents move beyond rote criticism to actionable guidance and progressively align their clinical performance with professional standards. Thematic evidence suggests that this structured dialogue facilitates the transition from passive learners to active, adaptive problem-solvers who engage in continuous self-reflection.

### Theoretical contributions: boundary-learning and adaptive performance

In this study, the boundary-learning framework functioned as an active operational guide, utilizing Mini-CEX and DOPS as “boundary objects” to bridge the gap between specialized knowledge and holistic generalist practice. In the residency context, “innovation” is best understood as adaptive clinical performance ‒ the transition from routine expertise to the flexible application of skills in multi-morbid environments. These findings suggest that receiving simultaneous feedback on interpersonal consultation and technical execution forces residents to navigate the cognitive friction at disciplinary intersections.

Furthermore, this approach aligns with the core objectives of General Medicine education reform, which shifts away from fragmented, specialty-based instruction toward an integrated, competency-centered model. By cultivating the ability to synthesize disparate clinical data within a unified management plan, the boundary-learning framework addresses the critical need for physicians who can manage multimorbidity in real-world settings. This transition from “siloed learning” to “boundary crossing” is essential for fostering a distinct generalist identity, ensuring that residents can provide high-quality, continuous care in increasingly complex healthcare systems.^11^^,^^15^

### Study limitations and future outlook

While the findings are encouraging, several methodological limitations must be acknowledged to contextualize the results: The prospective, single-arm design precludes a direct comparison with a control group. Therefore, score improvements likely encompass a combination of the intervention, the “testing effect” of repeated assessments, and the natural maturation of clinical experience over seven months. Regarding statistical transparency, the significant *F*-statistics from the repeated-measures ANOVA are consistent with the cumulative nature of skill acquisition. The robust effect sizes (Cohen’s *d* > 2.30) substantiate that the magnitude of improvement significantly exceeds typical measurement variability. The small cohort (*n* = 30) from a single center limits the generalizability of the findings. Additionally, this study relies on assessment scores as proxy indicators; the authors did not employ a direct, validated psychometric instrument to measure creativity or cognitive innovation. To build upon these insights, future research should employ multicenter Randomized Controlled Trials (RCTs) to disentangle the intervention's effects from natural skill acquisition. The authors also recommend developing specific rubrics to directly measure “clinical innovation” and using multivariate analysis to better control for confounders such as prior clinical experience.

## Conclusion

In conclusion, this exploratory pilot study suggests that an integrated Mini-CEX and DOPS assessment program is associated with measurable improvements in the clinical and procedural proficiency of General Medicine residents. However, these results should be interpreted as a proof-of-concept rather than a definitive confirmation of causal efficacy. Future multicenter Randomized Controlled Trials (RCTs) are essential to disentangle the specific effects of the boundary-learning framework from the natural progression of clinical experience and the effects of repeated assessment.

## Ethics approval

The study was approved by the Ethics Review Committee of Nantong University Sixth Affiliated Hospital and Yancheng Third People's Hospital in accordance with the principles outlined in the Declaration of Helsinki (Approval n° LS-2023–651). All participants were adults and provided written informed consent prior to their participation in the study.

## Funding

This work was supported by the 2024 College-local Collaborative Innovation Research Project of Jiangsu Medical College (202490138), the 2024 College-local Collaborative Innovation Research Project of Jiangsu Medical College (202490132), and the 2023 General Education Research Project of Kangda College, Nanjing Medical University (KDJYYJYB202315).

## Data availability statement

The data supporting the findings of this study are available from the corresponding author upon reasonable request.

## Declaration of generative AI and AI-assisted technologies in the writing process

During the preparation of this work, the author Shanshan Pang used generative AI and AI-assisted technologies (specifically Gemini) to perform a comprehensive spell-check and language polishing to improve the clarity, grammatical accuracy, and flow of the manuscript. Following this process, the authors reviewed and edited the content as necessary and take full responsibility for the integrity and accuracy of the final publication. All authors have reviewed and approved the final version of the manuscript.

## Conflicts of interest

The authors declare no conflicts of interest.
